# Systematic review of sporozoite infection rate of *Anopheles* mosquitoes in Ethiopia, 2001–2021

**DOI:** 10.1186/s13071-023-06054-y

**Published:** 2023-11-27

**Authors:** Yibeltal Aschale, Aklilu Getachew, Delenasaw Yewhalaw, Antonio De Cristofaro, Andrea Sciarretta, Getnet Atenafu

**Affiliations:** 1 Department of Medical Laboratory Sciences, Debre Markos University, Debre Markos, Ethiopia; 2 School of Medical Laboratory Science, Jimma University, Jimma, Ethiopia; 3 Department of Agriculture, Environment and Food Sciences, University of Molise, Molise, Italy; 4 Department of Biology, Debre Markos University, Debre Markos, Ethiopia

**Keywords:** *Anopheles* mosquito, Ethiopia, Sporozoite infection

## Abstract

**Background:**

Adult mosquitoes of the genus *Anopheles* are important vectors of *Plasmodium* parasites, causative agents of malaria. The aim of this review was to synthesize the overall and species-specific proportion of *Anopheles* species infected with sporozoites and their geographical distribution in the last 2 decades (2001–2021).

**Methods:**

A comprehensive search was conducted using databases (PubMed, Google Scholar, Science Direct, Scopus, African Journals OnLine) and manual Google search between January 1 and February 15, 2022. Original articles describing work conducted in Ethiopia, published in English and reporting infection status, were included in the review. All the required data were extracted using a standardized data extraction form, imported to SPSS-24, and analyzed accordingly. The quality of each original study was assessed using a quality assessment tool adapted from the Joanna Briggs Institute critical appraisal checklist. This study was registered on PROSPERO (International Prospective Register of Systematic Reviews; registration no. CRD42022299078).

**Results:**

A search for published articles produced a total of 3086 articles, of which 34 met the inclusion criteria. Data on mosquito surveillance revealed that a total of 129,410 anophelines comprising 25 species were captured, of which 48,365 comprising 21 species were tested for sporozoites. *Anopheles arabiensis* was the dominant species followed by *An. pharoensis* and *An. coustani* complex. The overall proportion infected with sporozoites over 21 years was 0.87%. Individual proportions included *Anopheles arabiensis* (1.09), *An. pharoensis* (0.79), *An. coustani* complex (0.13), *An. funestus* (2.71), *An. demeilloni* (0.31)*, An. stephensi* (0.70), and *An. cinereus* (0.73). *Plasmodium falciparum* sporozoites accounted 79.2% of *Plasmodium* species. Mixed infection of *Plasmodium vivax* and *P. falciparum* was only reported from one *An. arabiensis* sample.

**Conclusions:**

*Anopheles arebiensis* was the dominant malaria vector over the years, with the highest sporozoite infection proportion of 2.85% and an average of 0.90% over the years. Other species contributing to malaria transmission in the area were *An. pharoensis*, *An. coustani* complex, *An. funestus*, *An. stephensi*, and *An. coustani*. The emergence of new vector species, in particular *An. stephensi*, is particularly concerning and should be investigated further.

**Graphical Abstract:**

**Figure:**
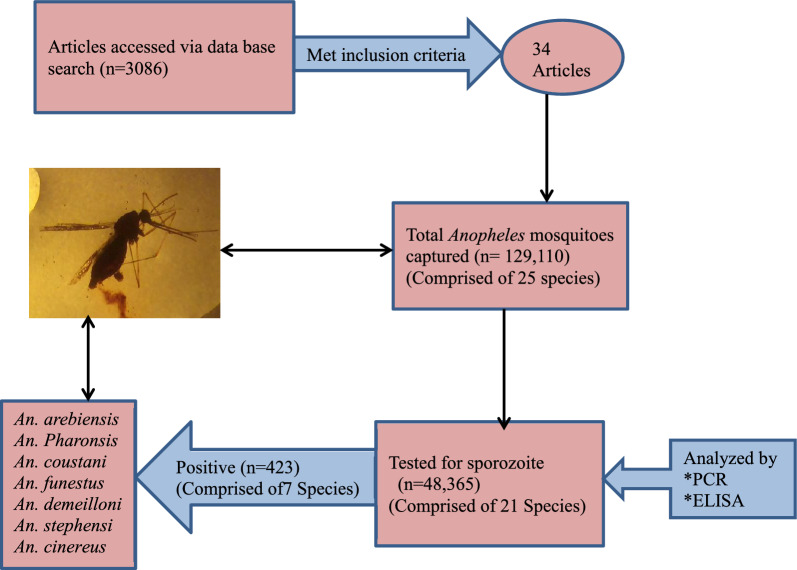

**Supplementary Information:**

The online version contains supplementary material available at 10.1186/s13071-023-06054-y.

## Background

Malaria is one of the most serious public health problems worldwide. In 2020, there were 241 million malaria cases (5.0% higher than 2019) and 627,000 deaths (34.8% higher than 2019) in 85 malaria-endemic countries worldwide [1]. *Plasmodium falciparum*, *P. vivax*, *P. ovale*, *P. malariae*, and *P. knowlesi* are the five *Plasmodium* species that cause malaria. Of them, *P. falciparum* and *P. vivax* are predominant and responsible for frequent mortalities and morbidities [2]. The disease transmission is aggravated by poverty and climate change [3–5].

Adult mosquitoes of the genus *Anopheles* are important vectors of *Plasmodium* parasites. Globally, there are over 500 identified species of *Anopheles* mosquitoes and approximately 70–80 transmit malaria in humans [6, 7]. In Africa, there are 140 *Anopheles* species and only 20 are known malaria vectors [8]. Their long-life span and strong human-biting behavior explain why over 90% of the world’s malaria cases are recorded from WHO African region each year [2]. More than 40 species of *Anopheles* mosquitoes have been identified in Ethiopia [9–11]. Of them, *Anopheles gambiae* complex is the primary vector of malaria, while *An. pharoensis*, *An. funestus*, *An. coustani* complex, and *An. nili* are secondary vectors [12, 13]. *Anopheles arabiensis* is the major vector species of the *An. gambiae* complex [14].

Recently, *An. stephensi* (the Asian malaria vector) has emerged on the African continent as a potential vector of both *P. falciparum* and *P. vivax*. It was first detected in Djibouti (2012) [15], Ethiopia (2016) [16], Sudan (2019) [17], Somalia (2019) [18], Nigeria (2020) [18], and most recently Kenya (2022) [19]. Detection was made morphologically and/or by sequencing recombinant DNA (rDNA) internal transcribed spacer region 2 (ITS2) and mitochondrial DNA cytochrome *c* oxidase subunit 1 (*cox*1) [16, 20].

Assessment of infection status of the malaria vectors in their salivary glands is crucial for vector incrimination and to estimate the number of infectious mosquitoes carrying *Plasmodium* sporozoites, which with human biting rate is important to evaluate entomological inoculation rate (EIR). *Plasmodium* sporozoite proportions can be estimated by detecting sporozoites under a microscope [21], using Circumsporozoite-ELISA (CS-ELISA) [22] or polymerase chain reaction (PCR) [23–25] from mosquito samples. In earlier times, microscopy was used to detect sporozoites in salivary glands for calculation of the percentage/proportion of infectious mosquitoes. However, this method requires fresh samples, more time, and skilled microscopists to dissect many mosquitoes, and it also fails to discriminate among *Plasmodium* species [26, 27]. Though CS-ELISA is the routinely used and widely accepted technique to detect sporozoites from naturally infected *Anopheles* mosquitoes, studies reported that it lacks specificity and overestimates the sporozoite rate (real infection) by detecting circumsporozoite protein in the hemolymph [23] and from the developing oocysts in the abdomen [21, 28].

Ethiopian malaria entomological data from 1930 to 2000 reported that the sporozoite infection rate of *An. arabiensis* ranges from 0 to 5.43% and the maximum sporozoite rate of *An. nili* was 1.57% [29]. In the last 2 decades (2001–2021), several entomological studies have been conducted to determine the infection rate of *Anopheles* mosquitoes by detecting *Plasmodium* CSPs using ELISA or any other methods (PCR and microscopy). However, no record is available on the overall and species-specific infection proportion of *Anopheles* species in Ethiopia. The present review aimed to summarize the past 21 years (2001–2021) overall and species-specific sporozoite infection proportion and regional distribution of *Anopheles* mosquitoes.

## Methods

### Search design and strategy

This systematic review has employed the Preferred Reporting Items for Systematic Reviews and Meta-Analysis (PRISMA) guidelines (Additional file 1: Table S1) [30]. The review protocol was registered in PROSPERO (International Prospective Register of Systematic Reviews; registration no. CRD42022299078). To find potentially relevant articles, a comprehensive search was conducted using electronic databases (PubMed, Google Scholar, Science direct, Scopus, African Journals OnLine) and manual Google search between January 1 and February 15, 2022. The search used the following search terms (MeSH and Text Words): ‘*Anopheles* species,’ ‘*Anopheles* mosquito,’ ‘malaria vector,’ ‘sporozoite infection,’ and ‘Ethiopia.’ The search terms were used in combination using Boolean operators like “OR” or “AND.” The reference lists of included studies were screened for additional eligible studies. Search results from different databases were exported to EndNote-X8 and then combined after removing duplicates.

### Study selection

Two researchers screened the selected articles for eligibility by reading the titles, abstracts, and then the full-text. Articles that did not report the outcome of interest were removed.

### Eligibility criteria

#### Inclusion criteria

Original articles conducted in Ethiopia, published in English between January 2001 and December 2021, reporting on female *Anopheles* mosquitoes of any species tested for circumsporozoite protein and sporozoite infection proportions.

#### Exclusion criteria

Mosquitoes other than *Anopheles* (e.g. *Culex* and *Aedes*) were not considered. Eligibility of each article was initially assessed based on titles and abstracts. Relevant articles were further evaluated using full texts. Studies without full texts were excluded after attempting to contact the primary author at least twice via email with no response. Additionally, studies lacking reported sporozoite infection statuses were excluded after reviewing their full texts.

### Data extraction

Two authors (YA and AG) extracted all the required data independently using a standardized data extraction form adapted from the JBI data extraction form for systematic reviews in a Microsoft Excel spreadsheet. Data were extracted from texts and tables of the original papers. Discrepancy between investigators on the data to be extracted was resolved via discussion or a third researcher. If still not resolved and additional clarification is required, the primary author of the included studies was contacted via email at least two times. Nominal and numerical data such as primary author, year of publication, study site, altitude of the study area, collection methods, number of *Anopheles* mosquito collected, *Anopheles* species identified, number of tested *Anopheles* mosquitoes, number of infected *Anopheles* mosquitoes, overall *Plasmodium* sporozoite rate, *Plasmodium* species detected, and methods used to detect sporozoite were extracted in Excel format.

### Assessment of methodological quality

Methodological quality and possibility of bias were assessed before inclusion in the review by undertaking critical appraisal to incorporate into the review process. Two authors (YA and AG) independently assessed the quality of each article using a standard quality assessment tool adapted from the JBI critical appraisal checklist for studies reporting prevalence data (Additional file 2: Table S2) [31]. Disagreements between two assessors were resolved via discussion and by contacting the primary author of the included studies via email. The tool has nine quality items that focus on sample size, methods used for identification of the outcome, and statistical analysis. Articles with a score ≥ 70% were considered high quality. Articles with a score between 69 and 51% and ≤ 50% were considered as moderate and poor quality respectively. None of the 34 articles was poor quality. The cut-off point was determined after reading other literature.

### Data processing and analysis

The data were extracted using Microsoft Excel 2010 spreadsheet and imported to SPSS version 24 software for analysis. The detailed description of the included original articles is presented on a table. Frequency and percentage were calculated to determine the overall and species-specific sporozoite infection proportion, frequency of occurrence, and to analyze regional distribution of anophelines. Mean and range were computed to describe the range and mean infection proportion of each *Anopheles* species over 21 years. The results were then presented in charts and tables.

## Results

### Literature search results

**Fig. 1:**
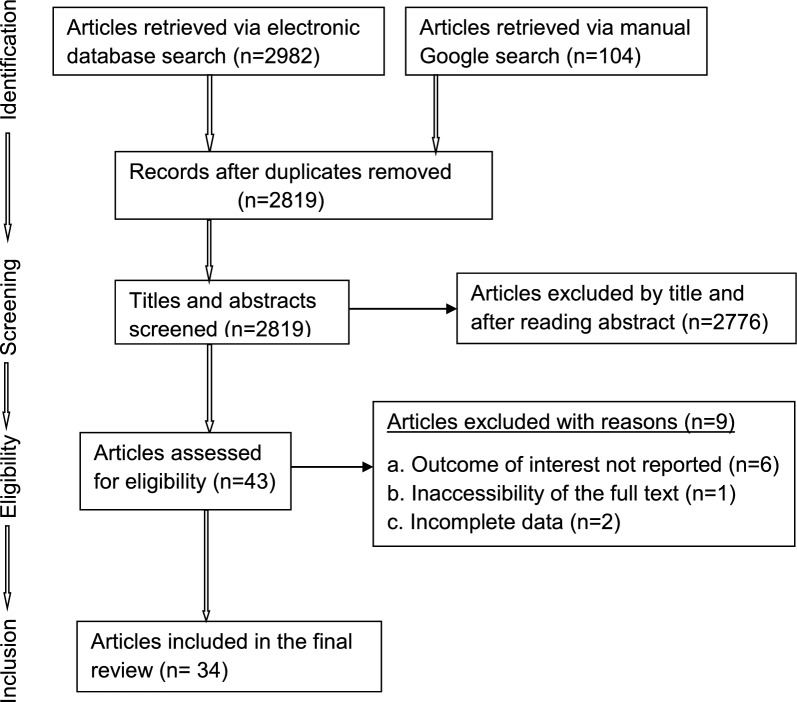
Flow chart of study selection for systematic review of sporozoite infection rate of *Anopheles* mosquitoes in Ethiopia, 2001–2021

The search of international databases and manual Google provided a total of 3086 published studies. After the removal of duplicates, 2819 remained. Of these, 2776 studies were discarded after reading their titles and abstracts since they did not meet the inclusion criteria. Of the 43 studies assessed for eligibility, one study was discarded because of the inaccessibility of the full text at the time of review and the full text of 42 studies was assessed. Then, six studies were discarded since they did not report the outcome of interest, and two studies were discarded because of incomplete data. Finally, 34 studies were included in the review process (Fig. 1).

### Study characteristics

All experiments included in this review were conducted based on standardized procedures and guidelines for CSP or DNA detection [6, 22, 25, 32–34]. The studies were published between January 1, 2001, and December 31, 2021, and conducted in different (western, eastern, central, southern, and northern) parts of Ethiopia. Twenty-eight studies were longitudinal studies, four were cross-sectional, and two were randomized control trials (Additional file 3: Table S3) [20, 35–67].

### Species diversity and frequency of *Anopheles* species

According to this review, 129,410 *Anopheles* mosquito specimens were captured from 2001 to 2021. A total of 25 *Anopheles* species have been recorded in different parts of Ethiopia, with an altitude ranging from 751 to 2660 m above sea level. They were collected from both indoor and outdoor sites. The collection methods employed were CDC light trap, human landing catch (HLC), pyrethrum spray catch (PSC), manual aspiration, artificial shelter, pitfall trap, clay pots, exit traps, human-baited double net traps, and cattle-baited traps (Additional file 3: Table S3). In all studies, sub-speciation using molecular testing has been performed on *An. gambiae* complex, and it has been confirmed to be *An. arabiensis*.

**Fig. 2:**
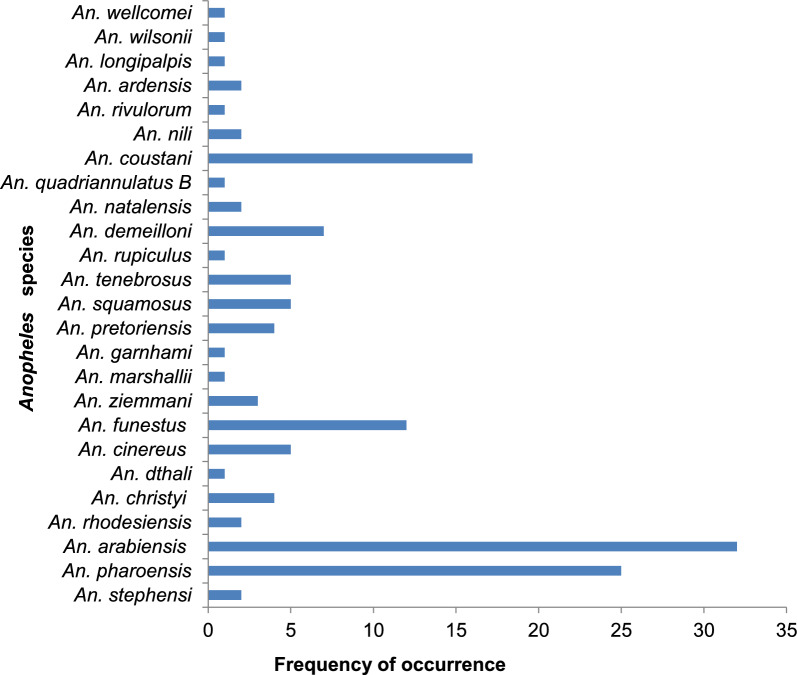
Frequency of *Anopheles* species in the four original studies from 2001 to 2021

*Anopheles arabiensis* comprised the highest frequency (recorded in 32 out of 34 studies), followed by *An. pharoensis* (recorded in 25/34 studies)*, An. coustani* complex (16/34), and *An. funestus (*13/34). *Anopheles stephensi* and *An. cinereus* have also been recorded in two (2/34) and one (1/34) study respectively (Fig. 2).

### Sporozoite infection proportions of the tested *Anopheles* mosquitoes

Of the 25 *Anopheles* species identified, 21 species had been tested for *Plasmodium* sporozoite or DNA. The overall *Plasmodium* sporozoite infection proportion ranged from 0.0 to 3.88%. A total of 48,365 (37.4%) *Anopheles* mosquito samples were tested for circumsporozoite protein or DNA among 129,410 *Anopheles* mosquitoes captured. Of these, CSP/DNA was detected in 423 (0.87%) samples using one of the three methods (CS-ELISA, PCR, or microscopy). Only seven *Anopheles* species (*An. arabiensis, An. pharoensis, An. stephensi, An. coustani* complex, *An. funestus*, *An. cinereus*, and *An. demeilloni*) were found positive for sporozoites. None of the *Anopheles dthali*, *An. rhodiensis, An. longipalpis*, and *An. wellcomei* was tested for *Plasmodium* sporozoite. *Anopheles arabiensis, An. pharoensis, An. coustani*, and *An. funestus* had sporozoite infection proportion of 1.09%, 0.79%, 0.13%, and 2.71% respectively. Though non-significant, there was a slight variation in sporozoite infection proportion of these three vectors over 21 years. *Anopheles arabiensis* varied between 0 and 2.85% (mean = 0.90%), *An. pharoensis* varied between 0 and 2.13% (mean = 0.42%), and *An. coustani* complex varied between 0 and 0.61% (mean = 0.18%) (Table 1).

**Table 1:** Overall and species-specific sporozoite infection proportions of *Anopheles* species tested for circumsporozoite protein/deoxyribonucleic acid from 2001 to 2021

No.	*Anopheles* species	Number tested	Number positive	Proportion infected with sporozoites (mean)
1	*An. squamous*	88	0	0.00
2	*An. pharoensis*	5422	43	0.79 (0.42)
3	*An. quadriannulatus B*	328	0	0.00
4	*An. natalensis*	2	0	0.00
5	*An. pretoriensis*	30	0	0.00
6	*An. demeilloni*	649	2	0.31 (0.09)
7	*An. rupicolus*	18	0	0.00
8	*An. funestus*	738	20	2.71 (0.48)
9	*An. cinereus*	411	3	0.73 (0.31)
10	*An. rhodesiensis*	0	–	–
11	*An. marshallii*	763	0	0.00
12	*An. garnhami*	45	0	0.00
13	*An. arabiensis*	31,293	341	1.09 (0.90)
14	*An. coustani* complex	6081	8	0.13 (0.18)
15	*An. ziemmani*	1513	0	0.00
16	*An. christyi*	104	0	0.00
17	*An. dthali*	0	–	–
18	*An. stephensi*	852	6	0.70 (0.70)
19	*An. tenebrosus*	15	0	0.00
20	*An. nili*	5	0	0.00
21	*An. rivulorum*	2	0	0.00
22	*An. ardensis*	5	0	0.00
23	*An. longipalpis*	0	–	–
24	*An. wilsonii*	1	0	0.00
25	*An. wellcomei*	0	–	–
	Overall	48,365	423	0.87 (0.73)

### Geographical distribution of tested *Anopheles* species

The majority of the tested anophelines were from central Ethiopia (39.2%), followed by southern Ethiopia (35.4%) and western Ethiopia (22.4%). The lowest numbers of anophelines were tested from eastern (1.8%) and northern (1.2%) Ethiopia. *Anopheles arabiensis* was recorded and tested in all parts of Ethiopia. *Anopheles stephensi* and *An. cinereus* were identified and reported as a vector of malaria in eastern and northern Ethiopia respectively.

### Geographical distribution of sporozoite-infected *Anopheles* species

**Fig. 3:**
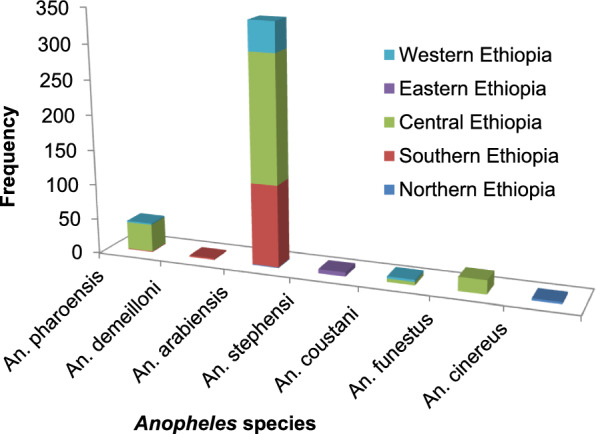
Distribution of sporozoite-infected *Anopheles* species in different parts of Ethiopia from 2001 to 2021

The highest numbers of sporozoite-infected *Anopheles* species were recorded in central Ethiopia (246/423), followed by southern Ethiopia (120/423). The fewest infected *Anopheles* species were recorded in northern Ethiopia (4/423). Infected *An. arebiensis* was recorded in all parts of Ethiopia except eastern Ethiopia. Sporozoite-infected *An. cinereus, An. stephensi*, and *An. demeilloni* were recorded in northern, eastern, and southern Ethiopia, respectively (Fig. 3).

### Frequency of sporozoite detection methods and species detected

**Fig. 4:**
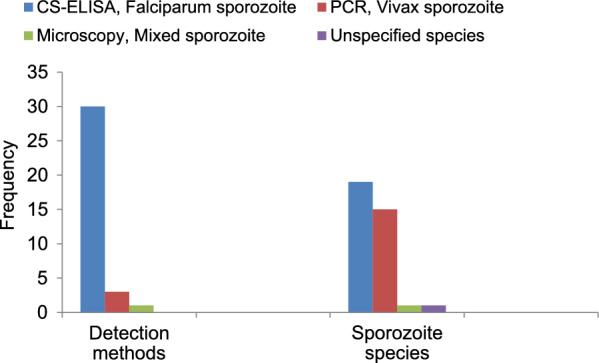
Frequency of sporozoite detection methods and sporozoite species detected from 2001 to 2021. CS-ELISA, circumsporozoite enzyme-linked immunosorbent assay; PCR, polymerase chain reaction

Overall, three detection methods (ELISA, PCR, and wet mount) were used in the 34 studies. Thirty studies (88.2%) used direct/sandwich ELISA, three studies (8.8%) used nested PCR, and one study (3.0%) used direct microscopy to detect *Plasmodium* sporozoites (Fig. 4).

*Plasmodium falciparum, P. vivax* (both *vivax*-210 and *vivax*-247 variants), and mixed sporozoites were detected from *Anopheles* samples tested positive. Either *P. vivax* or *P. falciparum* was isolated from examined anopheline samples in each study (Additional file 3: Table S3). *Plasmodium falciparum* sporozoites comprised the higher percentage (79.2%). Mixed infection of *P. vivax* and *P. falciparum* was only reported from one *An. arabiensis* sample (Fig. 4).

## Discussion

Vector control is a very effective approach to combat malaria transmission. Hence, it is an essential element of malaria control and elimination plans. The spread of native vectors, such as *An. stephensi*, into new areas is currently a challenge for WHO malaria elimination programs. Information about competent vectors of malaria with their geographical distribution is vital to public health and helps to mitigate and monitor the malaria threat in a certain area. For improved management and control of malaria, an update on the entomological profile is important to identify focus areas where vector surveillance is limited.

This review presented the previous 21-year (2001–2021) overall and species-specific sporozoite positivity rate and regional distribution profile of *Anopheles* species across Ethiopia. Twenty-five *Anopheles* species were recorded in different parts of the country with an altitude ranging from 751 to 2660 m above sea level. A previous review aiming to assess *Anopheles* species composition, abundance, and distribution reported a record of 35 *Anopheles* species in different parts of Ethiopia [68]. The difference in composition of *Anopheles* species is that the current study aimed to assess sporozoite infection proportion and included only studies that were conducted to determine infection status and reported sporozoite proportions.

Of the 129,410 *Anopheles* mosquitoes captured, 48,365 (37.4%) anophelines comprising 21 species were tested for circumsporozoite protein or parasite DNA using CS-ELISA and PCR techniques respectively from 2001 to 2021. This might be due to lack of resources and/or the remaining samples might not be eligible (nulliparous) for the determination of infection status which can be done via examination of abdominal status. The present review confirmed that *An. arabiensis, An. pharoensis*, *An. coustani* complex, *An. funestus, An. stephensi, An. cinereus*, and *An. demeilloni* were important malaria vectors of both *P. falciparum* and *P. vivax* in the area. Even though *An. nili* was incriminated as a vector of malaria in 1970 [69] and has been considered a secondary malaria vector in the Gambella area, none of the tested *An. nili* was sporozoite positive over 21 years.

According to the current review, the overall proportion of infected *Anopheles* mosquitoes from 2001 to 2021 was 0.87% with a mean overall proportion of 0.73%. Only seven species were found positive among 21 species tested. The sporozoite infection proportion of *An. arabiensis, An. pharoensis*, and *An. coustani* complex over 21 years ranged from 0 to 2.85% (mean = 0.90%), 0 to 2.13% (mean = 0.42%), and 0–0.61% (mean = 0.18%) respectively. The maximum sporozoite infection proportion of *An. arabiensis* in the present review was slightly lower than in a previous Federal Ministry of Health (FMoH) report (5.43%) [29]. *Anopheles funestus, An. cinereus*, and *An. demeilloni* had a mean sporozoite infection proportion of 0.48% (range 0–3.84%), 0.31% (range 0–0.92%), and 0.09% (range 0–0.58%) respectivly over 21 years.

Most the tested anophelines were from central Ethiopia (39.2%), followed by southern Ethiopia (35.4%). The fewest anophelines were tested from eastern (1.8%) and northern (1.2%) Ethiopia. This finding is similar to a previous review report focused on the abundance and distribution of *Anopheles* mosquitoes, which reported that the most abundant *Anopheles* mosquitoes were recorded in central Ethiopia and the fewest in eastern Ethiopia [68]. Similarly, the highest numbers of sporozoite-infected anophelines were recorded in central Ethiopia (*n* = 246) followed by southern Ethiopia (*n* = 120) compared to other areas, and the fewest sporozoite-infected anophelines were recorded in northern (*n* = 4) and eastern (*n* = 6) Ethiopia. Although several entomological studies were conducted in northern and eastern Ethiopia, they were focused on species composition, seasonal abundance, distribution, longevity, parous rate, and insecticide resistance despite the high prevalence of plasmodial infections among the population in these regions [70–74].

As shown in Fig. 4, the majority (88.6%) of studies have estimated sporozoite rates using CS-ELISA despite several limitations of this method. False-positive results have been reported by CS-ELISA compared with cytochrome b PCR (Cytb-PCR) [24]. A high rate of false positives due to cross-reactivity with non-*Plasmodium* antigens (host blood) has also been observed [75]. In Senegal, false-positive results were reported in *An. gambiae* sensu lato with bovine and/or sheep blood meals tested for the presence of *P. malariae* and *P. ovale* circumsporozoite protein [76]. In Thailand, false-positive results for *P. falciparum* and *P. vivax* have been found when testing the plasma fractions of pig and bovine blood [77].

Hasan et al. [24] revealed that a novel PCR method targeting *Plasmodium* mitochondrial cytochrome b (Mt Cytb-PCR) is highly sensitive and reliable for identifying *Plasmodium* species from *Anopheles* mosquitoes. It can detect the sporozoites as efficiently as CS-ELISA and nested PCR and detects low parasite density. Hendershot et al. [78] also confirmed that PCR-based method targeting mitochondrial cytochrome C oxidase subunit I (Mt COX‑I PCR) is highly sensitive in detecting *Plasmodium* DNA in mosquitoes. However, it is not a good candidate for sporozoite rate estimation because it detects DNA presence in all life stages of the parasite and hence has limited *Plasmodium* life-stage specificity. In this case, use of Mt COX‑I PCR together with CS-ELISA is recommended to confirm the real infection [78]. During sporozoite targeted DNA extraction and CS-ELISA assay, the head and thorax are commonly used, and infectious sporozoite stages are targeted from these organs [79, 80]. This helps to eliminate misreporting of positivity rates, which is mostly due to oocyst presence in the abdomen and/or rings,’ trophozoites,’ and gametocytes’ DNA in freshly ingested blood or incompletely digested blood.

Regarding the sporozoite species detected, the tested anophelines were infected with either *P. vivax* or *P. falciparum* sporozoites. Mixed infection of *P. vivax* and *P. falciparum* was only reported from one *An. arabiensis* sample. This supports the fact that *P. vivax* and *P. falciparum* are the predominant *Plasmodium* species causing malaria but are rarely present at the same time. Even though *P. ovale* and *P. malariae* cases have been reported in Ethiopia [81, 82], none of the tested samples were found infected with *P. ovale* and *P. malariae* sporozoites.

### Strength and limitations of the study

This is the first systematic review to our knowledge of the sporozoite infection proportion of *Anopheles* mosquitoes in Ethiopia conducted by reviewing original articles published in the last 2 decades. A recent systematic review by Adugna et al. [68] assessed only *Anopheles* species composition, abundance, and distribution in Ethiopia. However, our study might not provide an updated list of *Anopheles* mosquito species in Ethiopia since we have included only studies that were conducted to determine infection status and reported sporozoite proportions.

## Conclusions

The current review revealed that *An. arebiensis* has been the dominant malaria vector over the years, with the highest sporozoite infection proportion being 2.85% and an average of 0.90% over the study period. Other species contributing to malaria transmission in the area were *An. pharoensis, An. coustani* complex*, An. funestus, An. stephensi, An. cinereus*, and *An. demeilloni*. The emergence of new vector species, in particular *An. stephensi*, is particularly concerning and should be investigated further. Continued assessment of the specific role of different vector species is important to guide more effective control of malaria in the region. The use of head and thorax samples is recommended for sporozoite targeted DNA extraction and CS-ELISA assay to exclude contamination of oocyst sporozoites present in the abdomen. Moreover, *Anopheles* mosquito samples used for estimation of sporozoite infection proportion should be caught by human landing catch to avoid cross-reactivity since most mosquitoes that approach humans are expected to be unfed.

### Supplementary Information

**Additional file 1: Table S1**. PRISMA (Preferred Reporting Items for Systematic Reviews and Meta-Analysis) checklist for systematic review of sporozoite infection rate of *Anopheles* mosquitoes in Ethiopia, 2001–2021.

**Additional file 2: Table S2**. Methodological qualities of original studies included in the systematic review of sporozoite infection rate of *Anopheles* mosquito in Ethiopia, 2001–2021.

**Additional file 3: Table S3**. Descriptive summary of original studies included in the systematic review of sporozoite infection rate of *Anopheles* mosquito in Ethiopia, 2001–2021. CBT, cattle-baited trap; HDNT, human-baited double net traps; MA, mouth aspiration; PSC, pyrethrum spray sheet collection.

## Data Availability

The data supporting the findings of the study must be available within the article and/or its supplementary materials, or deposited in a publicly available database.

## Abbreviations

DNA: Deoxyribonucleic acid
PCR: Polymerase chain reaction
CSP: Circumsporozoite protein
ELISA: Enzyme linked immunosorbent assay
HLC: Human landing catch
JBI: Joanna Briggs Institute
