# Supplementary material for: Systematic review of sporozoite infection rate of Anopheles mosquitoes in Ethiopia, 2001–2021
Source: Parasit Vectors. 2023 Nov 27;16:437. doi: 10.1186/s13071-023-06054-y (PMC10680292; doi:10.1186/s13071-023-06054-y)
Supplement: Supplementary file 2 — Additional file 2: Table S2. Methodological qualities of original studies included in the systematic review of sporozoite infection rate of Anopheles mosquito in Ethiopia, 2001–2021. [file 13071_2023_6054_MOESM2_ESM.docx]

| **No** | **First author** | **Publication Year** | **Q1** | **Q2** | **Q3** | **Q4** | **Q5** | **Q6** | **Q7** | **Q8** | **Q9** |
| --- | --- | --- | --- | --- | --- | --- | --- | --- | --- | --- | --- |
| 1 | Tadesse et al | 2021 | Yes | NA | Yes | Yes | Yes | Yes | Yes | Yes | Yes |
| 2 | Tesfaye et al | 2011 | Yes | NA | Yes | Yes | Yes | Yes | Yes | Yes | Yes |
| 3 | Kibret et al | 2014 | Yes | NA | Yes | Yes | Yes | Yes | Yes | Yes | Yes |
| 4 | Getaneh et al | 2021 | Yes | NA | Yes | Yes | Yes | Yes | Yes | Yes | Yes |
| 5 | Taye et al | 2006 | Yes | NA | Yes | Yes | Yes | Yes | Yes | Yes | Yes |
| 6 | Animut et al | 2013 | Yes | NA | Yes | Yes | Yes | Yes | Yes | Yes | Yes |
| 7 | Gari et al | 2016 | Yes | NA | Yes | Yes | Yes | Yes | Yes | Yes | Yes |
| 8 | Habtewold et al | 2001 | Yes | NA | Yes | Yes | Yes | Yes | Yes | Yes | Yes |
| 9 | Kenea et al | 2016 | Yes | NA | Yes | Yes | Yes | Yes | Yes | Yes | Yes |
| 10 | Taye et al | 2016 | Yes | NA | Yes | Yes | Yes | Yes | Yes | Yes | Yes |
| 11 | Kibret et al | 2010 | Yes | NA | Yes | Yes | Yes | Yes | Yes | Yes | Yes |
| 12 | Lelisa et al | 2017 | Yes | NA | Yes | Yes | Yes | Yes | Yes | Yes | Yes |
| 13 | Massebo et al | 2013 | Yes | NA | Yes | Yes | Yes | Yes | Yes | Yes | Yes |
| 14 | Abraham et al | 2017 | Yes | NA | Yes | Yes | Yes | Yes | Yes | Yes | Yes |
| 15 | Degefa et al | 2015 | Yes | NA | Yes | Yes | Yes | Yes | Yes | Yes | Yes |
| 16 | Eba et al | 2021 | Yes | NA | Yes | Yes | Yes | Yes | Yes | Yes | Yes |
| 17 | Daygena et al | 2017 | Yes | NA | Yes | Yes | Yes | Yes | Yes | Yes | Yes |
| 18 | Getachew et al | 2019 | Yes | NA | Yes | Yes | Yes | Yes | Yes | Yes | Yes |
| 19 | Zemene et al | 2021 | Yes | NA | Yes | Yes | Yes | Yes | Yes | Yes | Yes |
| 20 | Dugassa et al | 2021 | Yes | NA | Yes | Yes | Yes | Yes | Yes | Yes | Yes |
| 21 | Degefa et al | 2021 | Yes | NA | Yes | Yes | Yes | Yes | Yes | Yes | Yes |
| 22 | Fettene et al | 2004 | Yes | NA | Yes | Yes | Yes | Yes | Yes | Yes | Yes |
| 23 | Aklilu | 2008 | Yes | NA | Yes | Yes | Yes | Yes | Yes | Yes | Yes |
| 24 | Kenea et al | 2019 | Yes | NA | Yes | Yes | Yes | Yes | Yes | Yes | Yes |
| 25 | Kibret et al | 2017 | Yes | NA | Yes | Yes | Yes | Yes | Yes | Yes | Yes |
| 26 | Getawen et al | 2018 | Yes | NA | Yes | Yes | Yes | Yes | Yes | Yes | Yes |
| 27 | Yewhalaw et al | 2014 | Yes | NA | Yes | Yes | Yes | Yes | Yes | Yes | Yes |
| 28 | Lemma et al | 2019 | Yes | NA | Yes | Yes | Yes | Yes | Yes | Yes | Yes |
| 29 | Kibret et al | 2012 | Yes | NA | Yes | Yes | Yes | Yes | Yes | Yes | Yes |
| 30 | Kindu et al | 2018 | Yes | NA | Yes | Yes | Yes | Yes | Yes | Yes | Yes |
| 31 | Nigatu et al | 2020 | Yes | NA | Yes | Yes | Yes | Yes | Yes | Yes | Yes |
| 32 | Balkew et al | 2021 | Yes | NA | Yes | Yes | Yes | Yes | Yes | Yes | Yes |
| 33 | Tirados et al | 2006 | Yes | NA | Yes | Yes | Yes | Yes | Yes | Yes | Yes |
| 34 | Bekele et al | 2012 | Yes | NA | Yes | Yes | Yes | Yes | Yes | Yes | Yes |

**Quality items Yes No NC NA**

1. Was the sample frame appropriate to address the target population?
2. Were study participants sampled in an appropriate way?
3. Was the sample size adequate?
4. Were the study subjects and the setting described in detail?
5. Was the data analysis conducted with sufficient coverage of the identified sample?
6. Were valid methods used for the identification of the condition?
7. Was the condition measured in a standard, reliable way for all participants?
8. Was there appropriate statistical analysis?
9. Was the response rate adequate, and if not, was the low response rate managed appropriately?

34

0

0

Overall appraisal: Include Exclude Seek further info
