# Supplementary material for: Systematic review of sporozoite infection rate of Anopheles mosquitoes in Ethiopia, 2001–2021
Source: Parasit Vectors. 2023 Nov 27;16:437. doi: 10.1186/s13071-023-06054-y (PMC10680292; doi:10.1186/s13071-023-06054-y)
Supplement: Supplementary file 3 — Additional file 3: Table S3. Descriptive summary of original studies included in the systematic review of sporozoite infection rate of Anopheles mosquito in Ethiopia, 2001–2021. CBT, cattle-baited trap; HDNT, human-baited double net traps; MA, mouth aspiration; PSC, pyrethrum spray sheet collection. [file 13071_2023_6054_MOESM3_ESM.docx]

|  | | | | | | | | | | |
| --- | --- | --- | --- | --- | --- | --- | --- | --- | --- | --- |
| **Author (Reference)** | **Study area** | **Study design** | **Collection methods** | **Number collected** | ***Anopheles spp* identified & tested** | **Number tested** | **Number Positive** | **Overall sporozoite rate** | ***Sporozoite spp* detected (N)** | **CSP detection Methods** |
| Tadesse et al, 2021[35] | Awash, Sebat kilo, eastern Ethiopia | Longitudinal | CDC, HLC, PSC, MA, CBT | 89 | *An.stephensi*  *An pharoensis*  *An*. Arabiensis | 72  1  16 | 3  0  0 | 3.37% | *P.vivax*(2)  *P.falciparum*(1) | Nested PCR |
| Tesfaye et al, 2011 [36] | Butajira, SNNP | Longitudinal | CDC, PSC, MA | 80 | *An.*arabiensis *An.christyi* | 44  36 | 0  0 | 0.0% | - | ELISA |
| Kibret et al, 2014 [37] | Ziway,  Central Ethiopia | Longitudinal | CDC | 5852 | *An.arabiensis*  *An.pharoensis*  *An.coustani*  *An.funestus* | 2335  1063  244  58 | 36  8  0  0 | 1.19% | *P.falciparum*  (44) | ELISA |
| Getaneh et al, 2021 [38] | Bahir Dar city, Northwest Ethiopia | Cross-sectional | CDC | 378 | An. arabiensis  *An.dthali*  *An.rhodesiensis* | 35  -  - | 1 | 2.85% | Species unspecified | Microscopy |
| Taye et al, 2006 [39] | Sille, Southern Ethiopia | Longitudinal | HLC | 1948 | *An arabiensis*  *An.pharoensis*  *An funestus*  *An.coustani*  *An.nili*  *An. marshallii* | 796  -  -  -  -  - | 18 | 2.26% | *P.falciparum* (4)  *P.vivax*(14) | ELISA |
| Animut et al, 2013 [40] | South Central Ethiopia | Longitudinal | CDC, PSC, Pit traps | 4558 | *An arabiensis*  *An.demeilloni*  *An.christyi*  *An.cinereus*  *An. pharoensis* | 819  119  61  39  79 | 16  0  0  0  2 | 1.61% | *P.falciparum* (2)  *P.vivax*(16) | ELISA |
| Gari et al, 2016 [41] | Adami Tulu, South central Ethiopia | Longitudinal | CDC  Artificial pit shelter  PSC | 232 | *An*. arabiensis *An.ziemmani*  *An.pharoensis*  *An.funestus* | 165  12  49  6 | 0  0  0  0 | 0.0% | - | ELISA |
| Habtewold et al, 2001 [42] | Gamo-Gofa, Southern Ethiopia | Longitudinal | MA | 278 | *An.arabiensis* | 262 | 3 | 1.15% | *P.falciparum* (3) | ELISA |
| Kenea et al, 2016 [43] | Adami Tulu, South Central Ethiopia | Longitudinal | HLC | 3,408 | *An*. arabiensis *An.funestus*  *An.ziemmani*  *An.pharoensis* | 800  60  1500  200 | 0  0  0  0 | 0.0% | - | ELISA |
| Taye et al, 2016 [44] | Jimma zone,  Southwestern Ethiopia | Longitudinal | CDC | 1,136 | *An*. arabiensis *An.coustani*  *An pharoensis* | 192  -  - | 0 | 0.0% | - | ELISA |
| Kibret et al, 2010 [45] | Ziway, Central Ethiopia | Longitudinal | CDC | 1271 | *An*. arabiensis  *An pharoensis*  *An.coustani* | 424  509  131 | 5  3  0 | 0.75% | *P.falciparum*(8) | ELISA |
| Lelisa et al, 2017 [46] | Kersa district,  Southwest Ethiopia | Longitudinal | CDC, PSC | 1559 | *An.* arabiensis *An.coustani*  *An pharoensis* | 192  -  - | 2 | 1.04% | *P.vivax*-210(2) | ELISA |
| Massebo et al, 2013 [47] | Chano, Southwest Ethiopia | Longitudinal | CDC, PSC, Pit shelter | 4708 | *An.* arabiensis  *An.marshallii*  *An.garnhami*  *An.funestus*  *An pharoensis*  *An.tenebrosus* | 3678  763  45  26  15  7 | 14  0  0  0  0  0 | 0.31% | *P.falciparum*  (11)  *P.vivax*-210 (3) | ELISA |
| Abraham et al, 2017 [48] | Sille, Southern Ethiopia | Longitudinal | CDC, Exit trap | 1291 | *An.*arabiensis  *An.pharoensis*  *An.pretoriensis*  *An.tenebrosus*  *An.rhodesiensis* | 872  184  -  -  - | 8  1 | 0.85% | *P.falciparum* (5)  *P.vivax*-210 (4) | ELISA |
| Degefa et al, 2015 [49] | Jimma town, Southwest Ethiopia | Longitudinal | CDC, PSC | 1912 | *An*. arabiensis *An.coustani*  *An pharoensis*  *An.squamosus* | 1271  164  57  18 | 23  1  0  0 | 1.59% | *P.falciparum*  (20)  *P.vivax*(4) | ELISA |
| Eba et al, 2021 [50] | Southwest Addis Ababa | Longitudinal | PSC | 2784 | *An*. arabiensis | 747 | 29 | 3.88% | *P.falciparum* (8), *P.vivax*(21) | Nested PCR |
| Daygena et al, 2017 [51] | Dirashe Woreda, South Ethiopia | Longitudinal | CDC | 1268 | *An.* arabiensis *An.demeilloni*  *An.cinereus*  *An.funestus*  *An. ardensis*  *An. pharoensis*  *An. christyi*  *An.pretoriensis*  *An. tenebrosus* | 755  347  -  -  -  -  -  -  - | 3  2 | 0.45% | *P.falciparum*(5) | ELISA |
| Getachew et al, 2019 [52] | Abeshge district, Southwest Ethiopia | Longitudinal | PSC, CDC, Pit shelter, MA | 2669 | *An*. arabiensis *An.coustani*  *An. demeilloni*  *An.rupicolus*  *An.nili*  *An. christyi*  *An.pretoriensis*  *An. tenebrous*  *An. rivulorum*  *An. ardensis*  *An. zeimanni*  *An. pharoensis*  *An.natalensis* | 1620  91  21  18  5  7  14  8  2  5  1  9  0 | 2  0  0  0  0  0  0  0  0  0  0  0  - | 0.11% | *P.vivax*(1)  *P.falciparum* (1) | ELISA |
| Zemene et al, 2021 [53] | Shebe Sambo district, Southwest Ethiopia | Longitudinal | HLC, PSC, CDC | 3659 | *An.coustani*  *An*. arabiensis  *An.pharoensis*  *An.squamosus* | 3088  415  128  28 | 0  0  0  0 | 0.0% | - | ELISA |
| Dugassa et al, 2021 [54] | Dangur district, Western Ethiopia | Longitudinal | HLC | 1970 | *An.* arabiensis  *An. demeilloni An.coustani*  *An.pharoensis*  *An.pretoriensis*  *An.natalensis* | 1702  87  80  42  16  2 | 9  0  0  0  0  0 | 0.47% | *P.falciparum*(8)  *P.vivax*-210(1) | ELISA |
| Degefa et al, 2021 [55] | Kersa district Jimma Zone, Southwest Ethiopia | Longitudinal | HLC,CDC,PSC,HDNT | 2038 | *An*. arabiensis  *An.pharoensis*  *An.coustani*  *An.squamosus*  *An.funestus* | 584  813  572  6  4 | 3  2  1  0  0 | 0.30% | *P.vivax*(3)  *P.falciparum*(3) | ELISA |
| Fettene et al, 2004 [56] | Southwestern Ethiopia | Longitudinal | PSC | 800 | *An*. arabiensis *An.quadriannulatus spp B* | 409  328 | 1  0 | 0.14% | Mixed (1) | ELISA |
| Aklilu, 2008 [57] | Koka area, Central Ethiopia | Longitudinal | CDC | 8279 | *An*. arabiensis  *An.pharonsis*  *An. squamosus* | 208  69  - | 0  0 | 0.0% | - | ELISA |
| Kenea et al, 2019 [58] | Adamitulu district, South central Ethiopia | Randomized Controlled Trial | CDC,PSC, HLC, pit shelter | 929 | *An.arabiensis* | 574 | 0 | 0.0% | - | ELISA |
| Kibret et al, 2017 [59] | Central Ethiopia | Longitudinal | CDC-light trap | 5238 | *An.arabiensis*  *An. Pharoensis*  *An.funestus*  *An.coustani*  *An. cinereus* | 2722  1596  521  399  0 | 79  27  20  0  - | 2.40% | *P.falciparum* | ELISA |
| Getawen et al, 2018 [60] | Arba Minch town, South west Ethiopia | Randomized controlled trial | CDC | 641 | *An.arabiensis*  *An. Pharoensis*  *An. tenebrous*  An*. longipalpis* | 562  0  0  0 | 16 | 2.85% | *P.vivax*(3)  *P.falciparum*  (13) | ELISA |
| Yewhalaw et al, 2014 [61] | Southwestern Ethiopia | Cross-sectional | CDC-Light trap | 520 | *An.arabiensis*  *An. coustani*  *An, demeilloni* | 461  37  22 | 4  1  0 | 0.96 | P.vivax | ELISA |
| Lemma et al, 2019 [20] | Northwest Ethiopia | Cross-sectional | CDC | 1221 | *An. cinereus*  *An. coustani*  *An. pharoensis*  *An. wilsonii*  *An. funestus*  *An. demeilloni* | 325  4  2  1  32  6 | 3  0  0  0  0  0 | 0.81% | *P.falciparum* | PCR |
| Kibret et al, 2012 [62] | South central, Ethiopia | Longitudinal | CDC | 2952 | *An.arabiensis*  *An. coustani*  *An. pharoensis*  *An. funestus* | 1634  879  410  29 | 16  5  0  0 | 0.71% | *P.falciparum* | ELISA |
| Kindu et al, 2018 [63] | Addis Zemen South Gondar, Ethiopia | Longitudinal | CDC, PSC, Pit shelter, clay pots | 182 | *An.arabiensis*  *An.cinereus*  *An. demeilloni* | 88  47  47 | 0  0  0 | 0.0% | - | ELISA |
| Nigatu et al, 2020 [64] | Northern, Southern, Central and Eastern Ethiopia | Longitudinal | CDC, HLC, PSC, Pitfall shelter, | 946 | *An.arabiensis*  *An. coustani*  *An.squamosus*  *An.pharonsis*  *An.funestus* | 86  214  36  9  2 | 0  0  0  0  0 | 0.0% | - | ELISA |
| Balkew et al, 2021 [65] | Eastern Ethiopia | Longitudinal | HLC, CDC, PSC and MA | 1040 | *An.stephensi* | 780 | 3 | 0.38% | *P.vivax* | ELISA |
| Tirados et al, 2006 [66] | Konso, Southern Ethiopia | Longitudinal | CDC, HLC, CBT, PSC | 63,194 | *An. arabiensis*  *An.pharonsis*  *An.funestus* | 6810  0  0 | 53  -  - | 0.78% | *P.vivax* (19)  *P.falciparum*  (34) | ELISA |
| Bekele et al, 2012 [67] | Adamitulu, East Shewa Zone, Central Ethiopia | Cross-sectional | CDC | 380 | *An. pharoensis*  *An. coustani*  *An. arabiensis*  *An. wellcomei* | 187  178  15  0 | 0  0  0  - | 0.0% | *-* | ELISA |
